# Invasive Mole of the Uterus: A Case Report

**DOI:** 10.7759/cureus.94005

**Published:** 2025-10-07

**Authors:** Alicja Dorota, Piotr Ficenes, Nicole Maryniak, Michal Dorota, Bartosz Czyzewski

**Affiliations:** 1 Medicine, Zaglebiowskie Oncology Center in Dąbrowa Górnicza, Dąbrowa Górnicza, POL; 2 Obstetrics and Gynecology, Zaglebiowskie Oncology Center in Dąbrowa Górnicza, Dąbrowa Górnicza, POL; 3 Conservative Dentistry, Central Clinical Hospital of the Medical University of Lodz, Łódź, POL; 4 Medicine, Central Clinical Hospital of the Medical University of Lodz, Łódź, POL

**Keywords:** beta-hcg, gestational trophoblastic disease, invasive mole, miscarriage, surgical treatment

## Abstract

This case presents a 31-year-old patient who was admitted due to secondary amenorrhea and high beta-human chorionic gonadotropin (hCG) levels. During hospitalization, a hydatidiform mole was identified. Initially, fractional curettage, hysteroscopy, and laparoscopy were performed. Due to the ineffectiveness of these methods, it was decided to perform a supracervical hysterectomy. Histopathological examination confirmed the presence of an invasive hydatidiform mole. hCG levels were monitored until they dropped to <1.2 mIU/ml. This case shows that surgical treatment can be an effective method for the patient if preservation of fertility is not necessary.

## Introduction

Gestational trophoblastic disease is a group of pregnancy-related conditions divided into hydatidiform moles (complete and partial) and gestational trophoblastic disease (invasive moles, choriocarcinoma, placental trophoblastic tumor, and epithelial trophoblastic tumor) [1]. Invasive mole is a trophoblastic disease, and it results from abnormal fertilization of the ovum [1]. There are two types of hydatidiform moles, complete diploid and partial triploid [2]. The incidence and risk of this condition depend on factors such as ethnicity, age, previous illnesses, and dietary errors [2-5]. Diagnosis is based primarily on high beta-human chorionic gonadotropin (hCG) levels and characteristic findings on diagnostic studies [6]. The treatment of choice is chemotherapy, with methotrexate being the most commonly used drug. Depending on the patient's preferences and clinical situation, multi-drug chemotherapy and surgical treatment are also used [6]. Beta-hCG levels should be monitored again 12 months after a negative result [7].

## Case presentation

**Figure 1:**
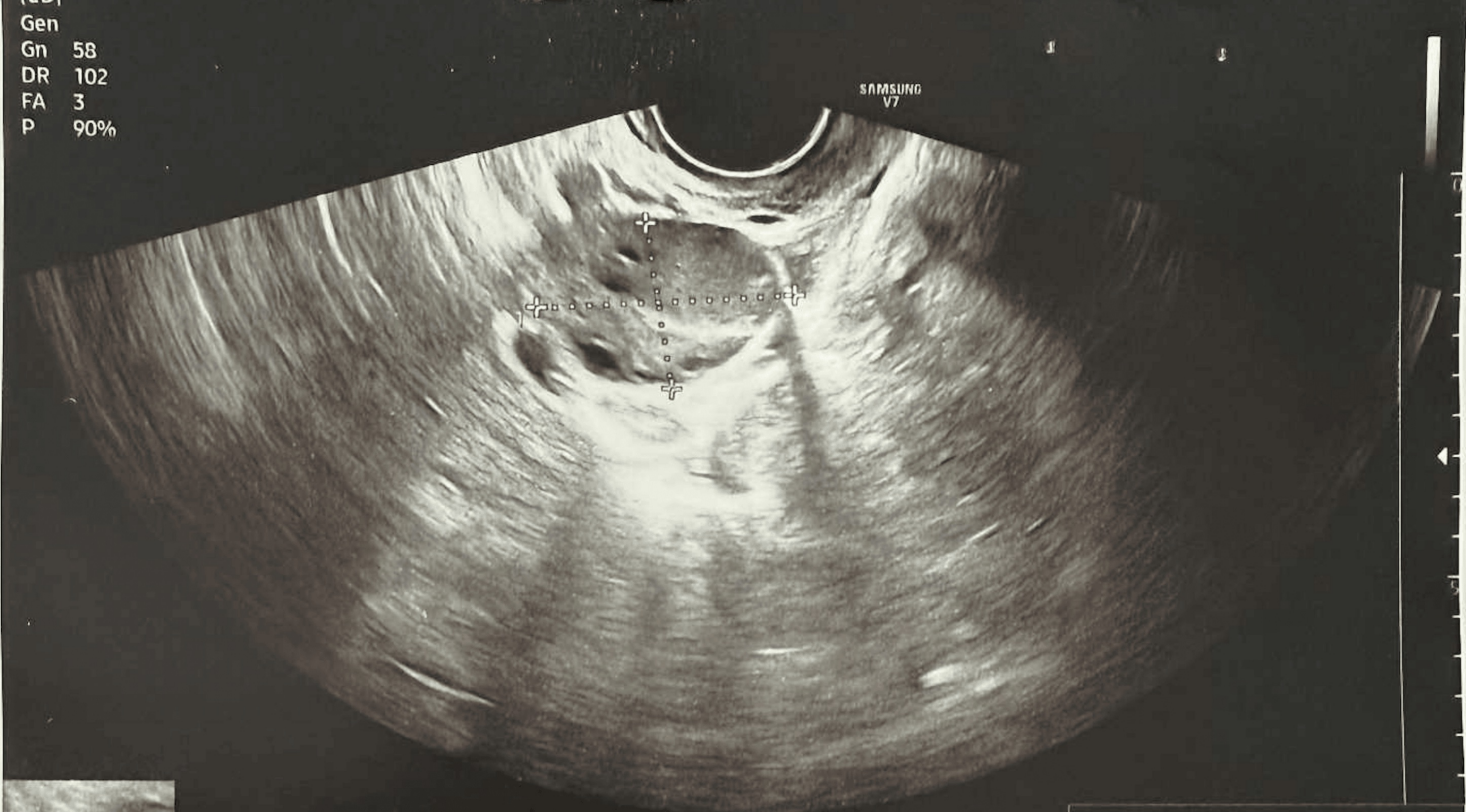
Ultrasound image from the day of patient admission showing a heterogeneous hyperechoic area

A 31-year-old woman was admitted to the Gynecology department due to secondary amenorrhea with suspected incomplete spontaneous abortion. Her medical records reported abnormal uterine bleeding and a history of insulin resistance. Her last menstrual period was 66 days ago. The patient had two children, both delivered via cesarean section. Ultrasound did not reveal a sac with an embryo in the uterine cavity but revealed a heterogeneous hyperechoic area near the left uterine horn, suggesting an ectopic pregnancy located in the intramural segment of the fallopian tube (Figure 1).

**Figure 2:**
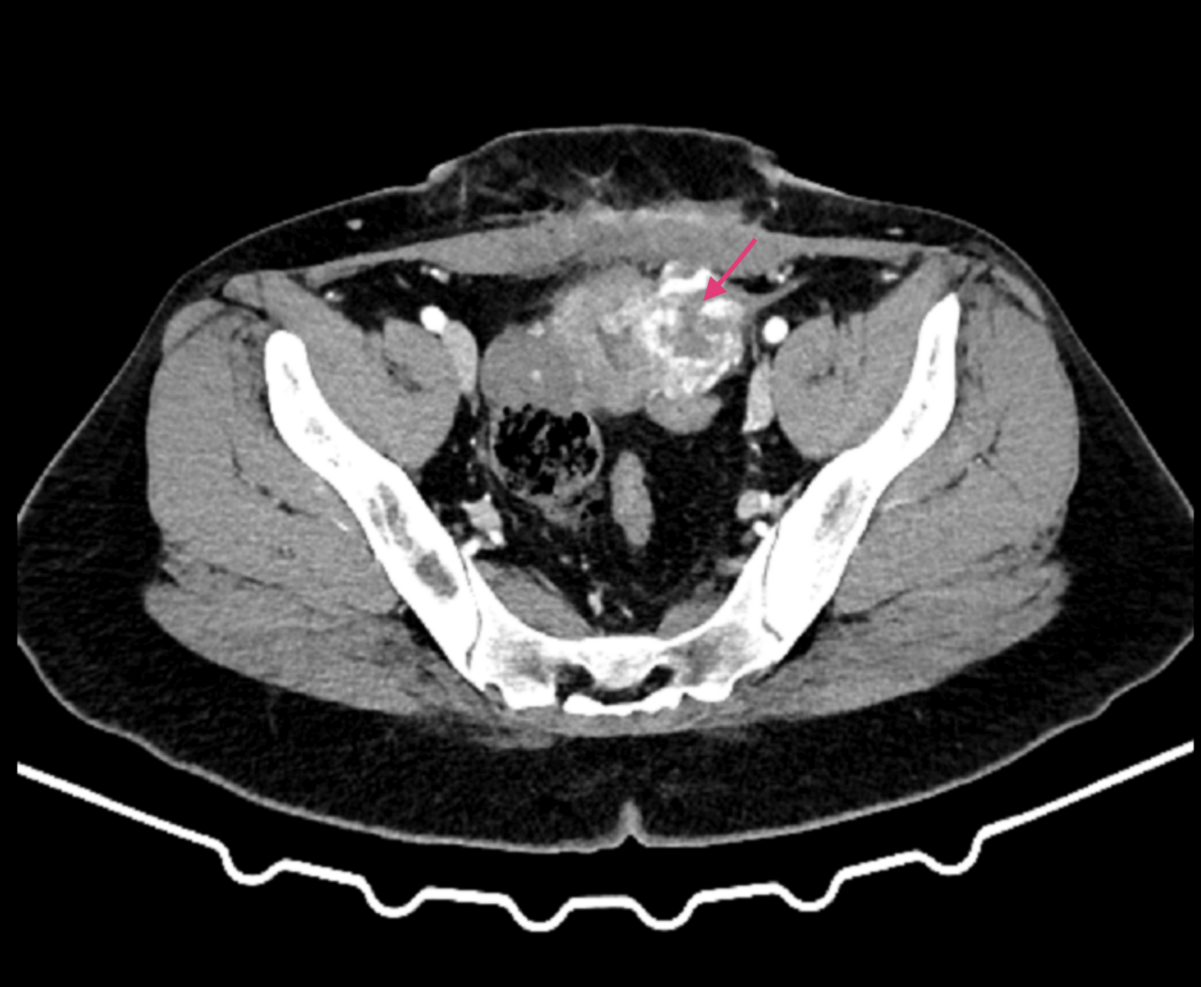
Computed tomography (CT) demonstrating a nodular mass in the uterine body (pink arrow)

After additional testing, the patient was scheduled for fractional curettage with tissue collection for histopathological examination. The procedure was uneventful, and the sample collected for testing was scant. Follow-up laboratory tests revealed that beta-hCG levels increased from 37,266.1 mIU/ml to 38,321.8 mIU/ml and 45,082.2 mIU/ml the following day. A decision was made to perform an exploratory laparoscopy to look for an ectopic pregnancy. Hysteroscopy was performed. During curettage, the posterior uterine wall perforated, resulting in conversion to laparotomy. Numerous adhesions were identified in the abdominal cavity; these were released, and the perforated uterine wall was sutured. Subsequent follow-up tests revealed beta-hCG levels reaching a maximum of 46,829.6 mIU/ml. A decision was made to perform a CT scan of the abdomen and pelvis with contrast agent. This revealed a nodular mass measuring 40x35 mm in the left lateral part of the uterine body, which enhanced intensely and heterogeneously, especially during the arterial phase of the post-contrast scan, with visible small foci of extravasated blood (Figure 2).

**Figure 3:**
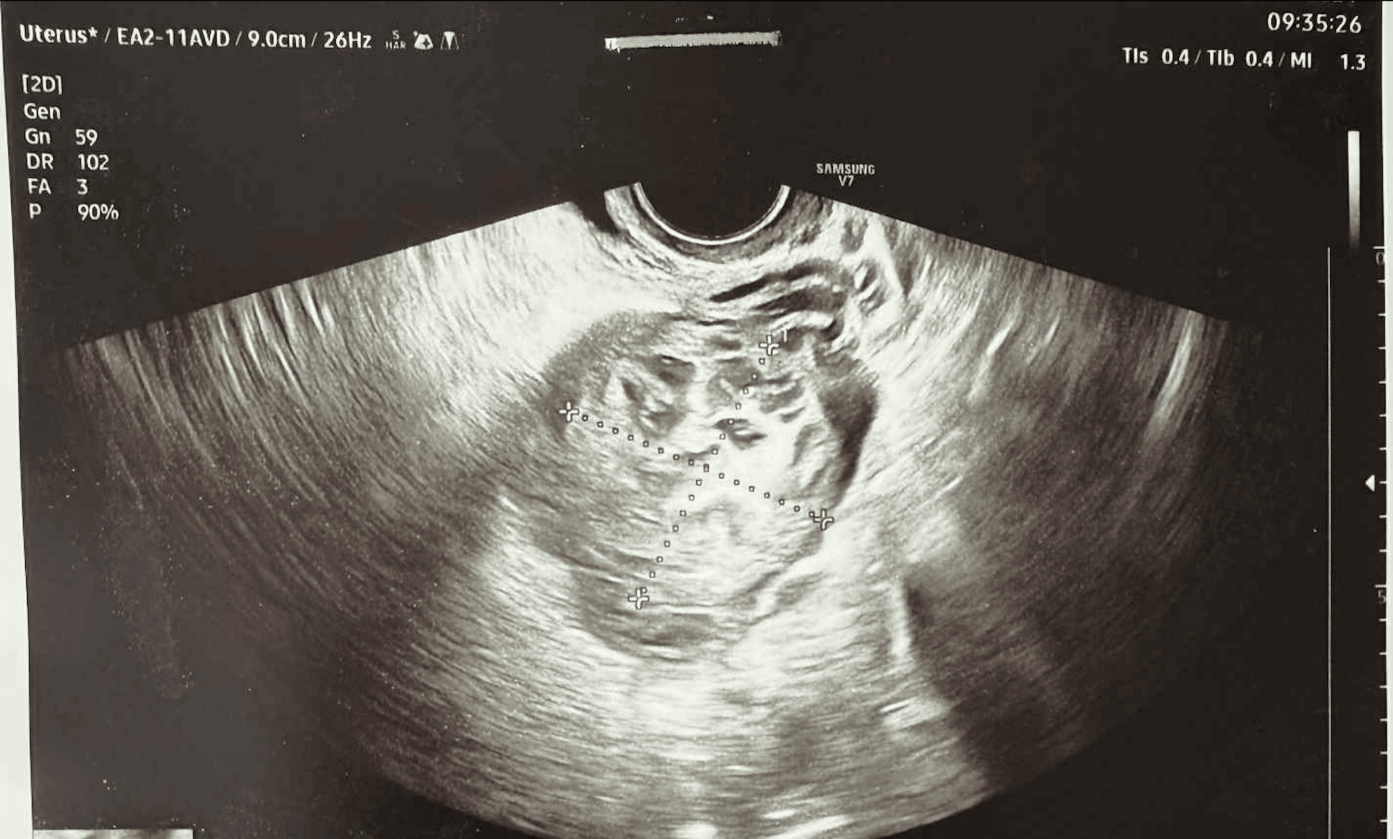
Progression of the lesion on ultrasound

Based on the CT scan and clinical data, a choriocarcinoma was suspected. Therefore, an oncology consultation was arranged, during which a CT scan of the chest, an MRI of the brain, and an attempt at uterine aspiration were recommended. Due to the emotionally difficult situation, a psychological consultation was arranged at the patient's request. After unsuccessful aspiration and an oncology reconsultation, the patient was offered surgical treatment. On follow-up ultrasound, a change in the characteristics of the lesion was observed as compared to the ultrasound performed at admission (Figure 3).

The patient denied any future plans for childbearing. After the patient consented, a relaparotomy with a supracervical hysterectomy, salpingectomy, and sampling of the ovaries was performed. Intraoperative examination revealed a non-specific neoplastic lesion, with no visible signs of cellular atypia or necrosis. The final histopathological examination, after paraffin examination, revealed an invasive hydatidiform mole, with disease limited to the endometrium and a maximum thickness of 2.5 cm. The day after surgery, the beta-hCG level dropped to 9634.8 mIU/ml and to 2312.7 mIU/ml the following day. The patient was discharged with recommendations and provided a beta-hCG test result of <1.2 mIU/ml. She was instructed to monitor her beta-hCG levels monthly for the next year and to report any increases.

## Discussion

Hydatidiform moles result from abnormal fertilization of the ovum and can be classified into two main types: diploid complete moles, which contain only paternal genetic material (46 XX, 46 XY), and triploid partial moles, which contain both maternal and paternal genetic material (69 XXY, 69 XXX, 69 XYY) [8]. The risk of progression from a complete mole to an invasive form or choriocarcinoma is approximately 15%, while for a partial mole, it is around 1.5%. Factors that increase the risk of progression include elevated beta-hCG levels, the presence of hyperreactio luteinalis, and prolonged uterine bleeding [9]. A hydatidiform mole is relatively rare, with incidence varying by region; in Europe, it occurs in about 1 in 1,000 pregnancies, in the USA, in 1 in 1,500 pregnancies, and in Southeast Asia and Japan, in 1 in 500 pregnancies [2].

Women with a history of hydatidiform mole, adolescents, and those over 36 years of age are at greatest risk of developing gestational trophoblastic disease [3]. Other contributing factors, though less significant, include belonging to certain ethnic groups, such as Asian, Indian, or African American populations, and having dietary deficiencies in vitamin A, animal proteins, and beta-carotene [4,5]. Genetic predisposition has also been identified, with some cases associated with a missense mutation in the NLRP7 gene located on chromosome 19q [10]. Common metastatic sites of invasive moles include the lungs, vagina, liver, and brain, with rare involvement of the epidural space or urinary bladder. Metastases typically spread through the bloodstream [11,12]. Histopathological confirmation is not required to initiate treatment; diagnosis is generally based on elevated beta-hCG levels and imaging studies [6].

The most common symptoms of hydatidiform mole include abnormal uterine bleeding, excessive uterine enlargement, hyperreactio luteinalis, severe vomiting, and pregnancy-induced hypertension during the first trimester [7]. Treatment typically involves fractional curettage, vacuum aspiration, or, in selected cases, surgical removal of the uterus. The use of pharmacological methods for uterine evacuation is generally discouraged due to safety concerns [6]. Curettage carries a higher risk of uterine perforation as compared to standard methods [7]. In patients over 40 years of age who do not plan further pregnancies, hysterectomy may be considered due to the increased risk of extramural invasion of trophoblastic disease [7]. Invasive moles are generally chemosensitive, making chemotherapy the preferred treatment modality.

The choice of chemotherapeutic regimen is guided by the FIGO (International Federation of Gynaecology and Obstetrics) prognostic score, which predicts the likelihood of resistance to single-agent chemotherapy. The FIGO classification categorizes gestational trophoblastic disease into four stages. Stage I corresponds to a tumor confined to the uterine corpus, while Stage II refers to a tumor confined to the genitalia but extending from the uterus into the adnexa or vagina. Stage III indicates the presence of disease in the lungs regardless of involvement in the reproductive organs, and Stage IV refers to all other metastases outside the lungs [12,13].

The FIGO/WHO scoring system evaluates multiple prognostic factors, including patient age, type of antecedent pregnancy, interval since the index pregnancy, pre-treatment serum hCG levels, tumor size, site and number of metastases, and prior chemotherapy history [12]. Each factor is assigned a score ranging from 0 to 4 points. Lower scores correspond to more favorable prognostic features, whereas higher scores indicate increased risk. The total FIGO/WHO score is combined with the FIGO stage, expressed as Roman numerals for the stage and an Arabic numeral for the total score, separated by a colon [12].

Patients with a FIGO/WHO score below 6 are considered low-risk and typically respond well to single-agent chemotherapy, such as actinomycin D, which is associated with fewer treatment failures compared to methotrexate [14]. If the response to initial therapy is inadequate, a transition from a single-agent to a multi-drug regimen is recommended [7]. Patients with a score of 6 or higher are generally treated with multi-agent regimens, the most common being EMA-CO (etoposide, methotrexate, actinomycin D, cyclophosphamide, vincristine) [15]. Individuals with an NLRP7 gene mutation may be counseled regarding reproductive options, including the use of donor eggs [6]. After completion of treatment, monthly monitoring of beta-hCG levels is advised for at least 12 months following normalization, and patients are recommended to use effective hormonal contraception during the first year post-treatment [7,16].

## Conclusions

Early detection of invasive moles is critical for optimizing patient outcomes. Prompt diagnosis allows for effective intervention and tailored management strategies. Chemotherapy remains the cornerstone of treatment, though surgical options may be appropriate in selected cases based on individual clinical circumstances and reproductive considerations. Systematic follow-up with regular monitoring of beta-hCG levels during the first year after treatment is essential for identifying any recurrence or metastatic spread at an early stage, ensuring timely adjustment of the therapeutic approach.
